# Social disadvantage, context and network dynamics in later life

**DOI:** 10.1007/s10433-023-00767-w

**Published:** 2023-05-27

**Authors:** Nan Feng

**Affiliations:** Department of Sociology, Cornell University, 347 Uris Hall, Ithaca, NY 14850 USA

**Keywords:** Social disadvantage, Network dynamics in later life, Between-within models, Contextual factors, Social connectedness

## Abstract

How do personal networks evolve as individuals age? To what degree do social disadvantage and contextual factors matter for network dynamics in later life? This paper answers these two questions based on egocentric network data of older adults over a ten-year period. Specifically, I use longitudinal and nationally representative data on 1,168 older adults from the National Social Life, Health, and Aging Project. I use between-within models to separate the within- and between-individual effects of sociodemographic characteristics and contextual factors on three aspects of social connectedness in later life: network size, frequency of contact, and proportion of kin. Patterns of network change vary among people of different races and ethnicities as well as educational levels. Black and Hispanic respondents have a significantly smaller network size and a higher average frequency of contact with confidants. Moreover, Hispanic respondents have a higher proportion of kin in the network, compared to White respondents. Similarly, older adults with less education have a smaller network size, higher frequency of contact and higher proportion of kin in their confidant networks compared to those who attended college. Older adults who have better mental health are more likely to have a higher frequency of contact and higher proportion of kin. When an older adult starts to work for pay, their frequency of contact with confidants tends to increase. Older adults living in neighborhoods with stronger social ties are more likely to have a larger network size, higher frequency of contact, and lower proportion of kin in their confidant network. The above results show that disadvantaged backgrounds and contextual factors are associated with certain less favorable network characteristics, which helps to explain the concentration of social disadvantage on certain populations.

## Introduction

Personal networks develop and evolve continuously throughout the life course (Wellman et al. 1997; Bidart and Lavenu 2005). Some scholars suggest that network size is bell-shaped as a function of age (Wrzus et al. 2013). On average, the size of the network initially increases with age, peaks at young adulthood, and decreases monotonically after middle adulthood (Carstensen 1995; Carstensen et al. 1999; Lang 2003). However, others claim that the network dynamics in later life can be more complex (Cornwell et al. 2021). To capture such complexities, one needs not only to examine the structure of older adults’ personal networks, but also to investigate the social backgrounds and contextual factors that help shape these networks. Life course transitions and changes in social context result in exceptionally high network turnover rates for older adults (Cornwell and Laumann 2015). However, peripheral ties are especially likely to dissolve as individuals age, as compared with inner ties (English and Carstensen 2014). This illustrates an important phenomenon, namely, that different types of social ties have different likelihoods of dissolving as the social context shifts. This motivates my research questions: How do the size and structure of personal networks evolve as individuals age? To what degree do social disadvantage and contextual factors matter for network dynamics in later life?

A well-connected and well-structured personal network contributes to an individual’s well-being. But people of different sociodemographic backgrounds have different chances of building and maintaining such a network. Empirical studies have shown a positive association between advantageous personal networks and other life outcomes, including lower mortality risk, and better physical health and emotional well-being (Litwin et al. 2020). The supporting role of personal networks becomes more substantial in later adulthood, as older adults tend to rely on close ties for social support, especially once their health substantially declines (Ducharme et al. 2011). Social disadvantage is associated with less desired network positions as well as less favored network characteristics. However, both these lines of research are faced with the problem of reverse causality. Is it that a good social relationship leads to advantage in health, living situation, and economic standing, or do those same factors contribute to both a good social relationship and other positive life outcomes? In other words, the success in social relationships and other life outcomes could be the result of some other social factors, such as favorable sociodemographic background or contextual factors. Moreover, social contexts and networks could co-evolve as adults age. One way of compensating for this is to incorporate existing social disadvantage indicators and dynamic contextual factors simultaneously in the analysis. Among the relevant factors, the residential, working, and health contexts are often considered to play a key role in shaping personal networks (Sharkey and Faber 2014). Therefore, I include all these factors in my models.

This paper has two main parts. First, I present how older adults’ personal networks change over a ten-year period. Specifically, I show how the size, composition, and frequency of contact change. These are defining factors of a network’s structure and function (Guadalupe and Vicente 2021). Second, I examine how disadvantaged backgrounds and shifts in contextual factors correlate with network features. By comparing the network features of disadvantaged groups and the reference group, one can see how pre-existing disadvantage can impact network size and structure. By including the contextual factors, one can see how contexts can alter network size and structure. I explore how networks are constrained by pre-existing social disadvantage but can be altered by the social contexts. In the meantime, one can also see how personal networks compensate for pre-existing disadvantages. To achieve the analysis goals, I apply between-within models. The between-individual effect compares network structure and size differences across social groups. The within-individual change shows how contextual factors and personal networks evolve for an individual across time. The results have significant implications on how social networks are associated with social inequality.

## Social disadvantage, context, and network dynamics in later life

There is still debate about how personal networks evolve during the aging process. On the one hand, social disengagement theory claims that as individuals grow older, they become less engaged with their network members (Cumming and Henry 1961). This theory indicates that both network size and frequency of contact decrease as adults get older. Along this line of research, socioemotional selectivity theory proposes that as older adults age, they tend to downsize their networks in a way which benefits emotional well-being (Gross 1998; Carstensen 2006). They prioritize the social connections which satisfy their emotional needs, instead of social ties that give them useful information or financial benefits (English and Carstensen 2014). On the other hand, some scholars advocate that personal networks remain relatively stable as individuals age (Atchley 1989; Cornwell et al. 2021). These scholars acknowledge the potential network turnover that older adults experience due to changes in social contexts. For instance, retirement, health decline, relocation, and adult children moving away can all fracture existing ties of older adults. However, these scholars suggest that older adults actively adjust for the loss of ties and make efforts to build new connections. As a result, older adults achieve homeostasis in network size (Cornwell et al. 2021).

The structure of older adults’ networks is as vital as the network size. Different types of networks, such as diverse networks, family-centered networks, friend-centered networks, and restricted networks, usually indicate different accessibility to social resources (Antonucci et al. 2014; Kim et al. 2017). Moreover, the type and structure of social networks are associated with health outcomes, such as life expectancy, health habits, and quality of life (Fiori et al. 2006; Litwin and Shiovitz-Ezra 2006; Kim et al. 2017; Litwin and Levinson 2018; Park et al. 2018; Ye and Zhang 2019; Choi and Jeon 2021; Guadalupe and Vicente 2021). More diverse networks are associated with better mental and physical health (Litwin and Shiovitz-Ezra 2006), while restrained networks are linked to inferior mental health (Kim et al. 2017). In older adulthood, family-based networks are prevalent and of great importance for older adults (Litwin et al. 2020). Older adults with family-based networks are less likely to experience depressive thoughts and are more satisfied with their quality of life (Litwin et al. 2020). This paper examines the size and structure of the confidant network because its members are essential for supporting older adults as age increases. I expand on work that has studied how age, contextual factors, and sociodemographic background are intertwined and impact the close ties of older adults. The answer to this question contributes to our understanding of the role of personal networks in aging and the corresponding consequences for social inequality.

Pre-existing social disadvantage is associated with less favorable network characteristics. Previous studies have used limited education and racial minority status as the key indicators of social disadvantage (Goldman and Cornwell 2018). Older adults with higher education are more likely to have their adult children as confidants despite geographical distance (Schafer and Sun 2021). Race and ethnicity affect the size and composition of an individual’s network. Some scholars suggest that Black Americans are more likely to have a smaller network with higher frequency of contact. Their networks tend to be more family-based than other racial groups (Ajrouch et al. 2001). Older Black American adults and those who did not attend college are more likely to experience instability in their relationships with their adult children (Goldman and Cornwell 2018). This might cause them to lose access to important resources and support since parent-child ties are essential for older adults.

Contextual factors are the elements of the social environment in which one is embedded, and/or which describe essential changes in the life course which affect one’s social interactions. Contextual factors including neighborhood environment, job entry, retirement, functional health decline, change in mental health status, and relocation can also lead to personal network changes (Vanhoutte and Hooghe 2012; Wrzus et al. 2013). Neighborhood environments have significant implications on social inequality in the US (Ludwig et al. 2013; Levy et al. 2020). The community in which an individual lives is closely linked to their income level, educational outcome, high risk behavior, delinquent activity, criminal involvement, and mental and physical health (Morenoff et al. 2001; Sampson et al. 2002; Sampson and Raudenbush 2004; Ludwig et al. 2013; Levy et al. 2020). Based on a randomized social experiment, Ludwig et al. (2013) found that moving from a disadvantaged neighborhood to a less disadvantaged neighborhood benefits both mental and physical health, which in turn contributes to a higher level of life satisfaction. Disadvantaged neighborhoods are more likely to experience higher rates of crime and homicide (Sampson and Raudenbush 2004). Social cohesion within a neighborhood is relevant to the networks of the residents. Disadvantaged neighborhoods are associated with smaller network size for older adults (York Cornwell and Behler 2015). Older male adults who live in disadvantaged neighborhoods tend to have less frequent contact with family and friends (York Cornwell and Behler 2015). The influence of residential contexts on individuals’ personal networks persists across different life stages (Sharkey and Faber 2014).

Working status also impacts network composition and structure (Ajrouch 2005). Retiring adults are less likely to maintain co-worker relationships, which results in a decline in network size and a more closely knit network (Van Tilburg 2003). The closeness of social ties from work and the timing of retirement both impact how the network changes. Peripheral ties have a higher a probability to dissolve during the transition to retirement, regardless of occupational type (Van Tilburg 2003; Kauppi et al. 2021). Some scholars suggest that networks become more stable after the transition to retirement. Other life-course changes, such as mental health decline, functional health decline, and the transition into caregiving roles also affect how networks change in the long term (Perry and Pescosolido 2012; Roth 2020). For older adults, transitioning into caregiving roles might put a lot of strain on their psychological well-being and lead to conflicts with family members (Ducharme et al. 2011). Apart from impacting personal networks directly, social context can also be a moderating factor between networks and other life outcomes (Birditt et al. 2014).

The studies above provide us with broad knowledge of the essential factors shaping personal networks. As mentioned above, I take a dynamic perspective and track the changes in the contextual factors as well as the changes in personal networks across time. Social networks are dynamic in nature and the stability of networks differ for different social groups. Likewise, contextual factors can be unstable, especially for disadvantaged groups (Desmond 2012). Moreover, research in this area often focuses either solely on social disadvantage or contextual factors. Pre-existing social disadvantages and contextual factors may be closely intertwined. Individuals from disadvantaged social backgrounds have higher likelihood to be trapped in less favorable social environments and encounter more instability and insecurity throughout the life course (Desmond 2012; Goldman and Cornwell 2018). As a result, pre-existing disadvantage and the evolving contextual factors together shape the personal network of an older adult. Including both pre-existing social disadvantages and social contexts in the analysis allows one to distinguish between the impacts due to each separate factor. Examining the co-evolution of social environments and personal networks can help solve the problem of reverse causality. Furthermore, instability in personal networks can affect individuals’ health and socioeconomic outcomes. Some research has shown how various life events impact network size and structure over an extended time period. Despite the innovation and advancement in these studies, most are only based on a small number of observations which are not necessarily representative for an entire nationwide social group. My study contributes to this line of inquiry by analyzing changes in personal networks and their association with evolving contextual factors as well as pre-existing social disadvantage, based on longitudinal and population-based data.

I claim that network change is intertwined with pre-existing social disadvantage and contextual factors. On the one hand, people from disadvantaged backgrounds are more likely to rely on resources provided by their close social circles. Thus, they have the motivation to sustain existing network ties and seek potential new connections. On the other hand, individuals from underprivileged backgrounds might have limited resources to maintain or construct social ties. Previous theories, such as social convoy theory, suggest that close ties are less likely to dissolve than peripheral ties (Antonucci and Akiyama 1987; Van Tilburg 1992, 2003; Ajrouch et al. 2018). Socioemotional selection theory also emphasizes the value of close ties and how older adults make great efforts to preserve these ties. Although close ties are more enduring than peripheral ties, they can still be highly dynamic in later life (Cornwell and Laumann 2015; Cornwell et al. 2021). By using longitudinal and nationally representative data on older adults’ confidant networks, this paper captures the change in close ties over a 10-year period in later life. Furthermore, I examine how social disadvantage, contextual factors, and changes in these elements are associated with the change of network size and structure over a long time period in later life. Based on previous research, I propose the following hypotheses:

### Hypothesis 1

On average, network size, frequency of contact, and proportion of kin tend to decrease over the long term as age increases.

### Hypothesis 2

For older adults from disadvantaged backgrounds, personal networks tend to be smaller in size with a lower level of diversity.

### Hypothesis 3

Changes in the contextual factors can alter network size and structure, even after controlling for pre-existing social disadvantage.

## Data and methods

### NSHAP data

This study uses three rounds of data on 1,168 older adults from the National Social Life, Health, and Aging Project (NSHAP). NSHAP is the first nationally representative dataset on community-dwelling older adults egocentric network change in the United States. This study collected detailed information from older adults, including sociodemographic backgrounds, egocentric networks, marital and sexual relationship history, health, and neighborhood environment. This paper mainly utilizes the network section and sociodemographic background section. The first round of data was collected in 2005/2006 with a sample size of 3,005. The second round and third round of data were collected in 5-year increments. At Round 1, the older adults were aged between 57 and 85 years old. The sample of this study consists of older adults who were surveyed in all three rounds. The weighted and conditional response rates for Round 1, Round 2, and Round 3 are 75.5%, 89%, and 89.2%, respectively (Cornwell et al. 2021).

### Measures

The outcome variables are network size, frequency of contact, and proportion of kin. The confidant network is an egocentric network with the respondent as the center of the network. These egocentric networks were elicited by a widely used name generator. Specifically, each respondent was asked to give a list of names of people with whom they discussed important matters in the past 12 months.^1^ Three aspects of the egocentric confidant network are of most interest in this paper, namely, network size, average contact frequency, and network kin composition. Network size is the number of close contacts that older adults have in the egocentric network. The frequency of contact captures how often the ego talks to the alters on average. The proportion of kin is the percentage of kin members in respondent’s confidant network.

The explanatory variables can be divided into two groups: sociodemographic characteristics and contextual factors. For sociodemographic characteristics, I focus on respondent’s race and ethnicity and educational attainment. These two variables are often used to identify social disadvantage. For race and ethnicity, I generated three dummy variables with White respondents as the reference group. Racial groups and educational attainment are treated as time-invariant variables. For contextual factors, I primarily examine respondent’s functional and mental health status, working status, and neighborhood ties. Ideally, all the contextual variables should be time-varying variables. However, some variables are only available in a certain round of the survey. For instance, neighborhood ties are only collected at Round 2. It is still meaningful to include these screenshots of context to present a whole picture of how contextual factors impact network dynamics. Other contextual variables are all treated as time-varying variables in the models. Functional health, mental health, and working status were collected at all three rounds. The range for self-rated mental health is from 1 to 5,^2^ with larger scores indicating better mental health. Functional health is evaluated by respondent’s difficulty in ADL and IADL activities. The range of the functional health score is from − 27 to 0.^3^ 0 represents that there is no difficulty in ADL and IADL activities, while more negative values represent more difficulty in the ADL and IADL activities. Working status is a binary variable, documenting whether the older adult worked for pay in the last week.

Control variables include gender, age, marital status, and household size. Previous research has suggested networks and their change could differ among people of different gender, age, marital status, and household size. Based on the design of the NSHAP survey, gender is treated as a time-invariant variable. Age, marital status, and household size are treated as time-varying variables. Age is scaled by 10 to demonstrate the coefficient of age more clearly, and to show the differences between 10-year age groups more straightforwardly. Household size measures how many people live in the household being interviewed, including the respondent.

### Methods and models

The main model that I utilized is the between-within model (Allison 2009; Schunck and Perales 2017). I use some key social disadvantage indicators and contextual indicators to predict the change in network size, frequency of contact, and network composition. Three models were estimated using the explanatory variables and controls to predict the change in network size, the frequency of contact, and network kin composition in later life, respectively. I estimated the following between-within model:

$$y_{it} = a_{i} + \mathop \sum \limits_{m = 1}^{M} \beta_{W,m} \left( {x_{it,m} - \overline{x}_{i,m} } \right) + \mathop \sum \limits_{m = 1}^{M} \beta_{B,m} \overline{x}_{i,m} + \mathop \sum \limits_{k = 1}^{K} \gamma_{k} c_{i,k} + e_{it}$$

where ${y}_{it}$ is the network characteristic, ${x}_{it,m}$ is individual $i$’s explanatory variable $m$ at time $t$, ${\overline{x}}_{i,m}$ is individual $i$’s average value of variable $m$ over ten years, ${\beta }_{W,m}$ are the within-individual estimators, ${\beta }_{B,m}$ are the between-individual estimators, and ${c}_{i,k}$ is a set of control variables. The coefficient ${\beta }_{W,m}$ means each unit of within-individual change in explanatory variable $m$ is linked to ${\beta }_{W,m}$ change in the outcome variable ${y}_{it}$. One strength of between-within models over OLS models is that they separate within-individual effects and between-individual effects. This advantage is especially valuable for the time-varying explanatory variables that I study. The within-individual estimator captures how changes in a feature of an individual over ten years are associated with changes in network structure.

## Results

Table 1 shows the descriptive statistics of the variables in the models. The upper panel of the table shows the concentration tendency of the sociodemographic variables. 76% of the respondents are White. Black, non-Black Hispanic,^4^ and other race and ethnicity constitute 12%, 9%, and 2% of the sample respectively. 37% of the older adults only graduated from high school or less. On average, the mean of the self-rated mental health score is 3.9, per the range of 1–5. At Round 1, the average score of functional health is -1.4. This suggests that the respondents have moderate difficulty in ADL and IADL activities, on average. Over ten years, mental health scores remain relatively stable, while functional health declines monotonically. At Round 1, 40% of the respondents worked for pay recently. The average household size at Round 1 is 2, meaning that, on average, older adults live with at least one other person. The average size of older adult’s confidant network at Round 1 is 3.65. This number increases slightly to 3.92 five years later and remains steady in the following five years. On average, older adults talk to network members more than once a week. The frequency of contact decreased slightly in the following 10 years. At Round 1, 67% of the network members are kin. The composition of kin in confidant network decreases in the next 10 years, on average. As older adults get older, their confidant networks tend to shrink more. At the same time, the frequency of contact and proportion of kin tend to decrease.

**Table 1:** Sociodemographic variables, contextual variables, network characteristics and change (*N* = 1,168). *Source*: NSHAP

	R1 (Mean/%)	R2 (Mean/%)	R3 (Mean/%)
Age	66	71	76
*Gender (%)*			
Male (ref.)	46	46	46
Female	54	54	54
*Race/Ethnicity*^a^* (%)*			
White (ref.)	76	76	76
Black	12	12	12
Hispanic, non-Black	9	9	9
Other	2	2	2
*Education (%)*			
College or more (ref.)	63	63	63
High school graduates or less	37	37	37
*Marital status (%)*			
Not married or no partner present (ref.)	29	33	40
Married or partner present	71	67	60
*Health*			
Mental health score	3.9	3.8	3.9
Functional health score	− 1.4	− 1.6	− 2.7
Working status (%)			
Did not work for pay lately (ref.)	60	73	85
Worked for pay lately	40	27	15
Neighbor ties		4.6	
Household size	2	2	2
*Network characteristics*			
Network size	3.65	3.92	3.95
Frequency of contact	6.88	6.75	6.65
Proportion of kin	.67	.66	.63

^a^In the sample of 1168, no older adults reported changes in their race and ethnicity or gender or educational level over the ten-year period

My first research question asks how networks change as age increases. The between- and within-individual coefficients of age in Table 2 show that as an individual ages, the size of the social network increases while the frequency of contact and proportion of kin decrease. But if the results from the lower panel of Table 1 are combined, one can see that for the entire sample, the absolute change in network size, frequency of contact, and proportion of kin is relatively small. Based on Table 1, network size has a small increase while the frequency of contact and proportion of kin decrease monotonically for the entire sample, on average. Hypothesis 1 is partially supported. In addition, none of the between-individual coefficients are statistically significant. This indicates that no cohort difference in network size and structure is observed. However, this might be due to the small sample size of each cohort in the overall sample.

**Table 2:** Between-within models of network change, unstandardized coefficients, and standard errors (*N* = 1,168). *Source*: NSHAP

	Network size	Frequency of contact	Proportion of kin
	Model 1 (COEF/SE)	Model 2 (COEF/SE)	Model 3 (COEF/SE)
*Race/Ethnicity (ref. White)*			
Black	− .095***	.330***	.008
	(.029)	(.059)	(.023)
Hispanic, non-Black	− .112***	.342***	.088***
	(.033)	(.068)	(.026)
Other	.008	.054	.033
	(.059)	(.129)	(.049)
*Education (ref. College or more)*			
High school graduates or less	− .100***	.256***	.078***
	(.019)	(.041)	(.016)
*Mental health*			
Between individuals	.006	.108***	.023*
	(.013)	(.027)	(.011)
Within individuals	.008	.045*	− .004
	(.016)	(.021)	(.007)
*Functional health*			
Between individuals	.007	− .009	− .005
	(.004)	(.008)	(.003)
Within individuals	.004	.005	− .004
	(.005)	(.007)	(.002)
*Worked for pay lately*			
Between individuals	− .015	.081	− .018
	(.028)	(.060)	(.023)
Within individuals	− .034	.151***	.019
	(.032)	(.043)	(.015)
Neighbor ties	.010*	.026**	− .012***
	(.004)	(.009)	(.003)
*Control Variables*			
Female (ref. Male)	.128***	.036	− .010
	(.019)	(.040)	(.015)
Age * 10			
Between individuals	− .006	− .023	.020
	(.015)	(.032)	(.012)
Within individuals	.072**	− .171***	− .039***
	(.024)	(.032)	(.011)
*Married or partner present*			
Between individuals	.007	.097	.112***
	(.024)	(.052)	(.020)
Within individuals	− .004	.036	.051*
	(.049)	(.065)	(.023)
*Household size*			
Between individuals	.005	.094***	.027**
	(.013)	(.027)	(.010)
Within individuals	.010	.037	-.002
	(.015)	(.020)	(.007)
Constant	1.305***	5.907***	.312**
	(.126)	(.267)	(.102)
AIC	12760.736	8379.334	1233.609
BIC	12877.807	8508.729	1363.004
Number of observations level 2	1,168	1,168	1,168
Number of observations level 1	3,504	3,504	3,504

^*^p < .05; **p < .01; ***p < .001

When performing more detailed analyses by social groups, I find statistically significant differences between people from different social groups. The upper panel of Table 2 shows the association between pre-existing social disadvantage and network size and structure. As suggested beforehand, racial minority status and lack of college education are indicators of social disadvantage. In the upper panel of Table 2, the coefficients suggest that older Black and Hispanic adults tend to have a smaller network size than older White adults. At the same time, older Black and Hispanic adults tend to have higher frequency of contact. Older Hispanic adults tend to have higher proportion of kin in the confidant network. These findings align with previous studies. Lower educational level is associated with a smaller network size, more frequent contact, and higher proportion of kin in the confidant network. These results suggest that people from disadvantaged groups tend to have smaller networks, a higher frequency of contact, and are more likely to have kin-centered networks. Hypothesis 2 is partially supported. Previous literature has suggested that a larger network size and a more diverse network are more beneficial. My findings suggest that older adults from disadvantaged socioeconomic backgrounds are less likely to have these beneficial networks. However, pre-existing disadvantages do not dictate the size and structure of individuals’ networks over a long-time period. During older adulthood, as contextual factors shift, network features also shift.

Contextual factors play an important role in shaping older adults’ networks, net of pre-existing social disadvantage. Mental health is an important indicator of network change. Better mental health is associated with more frequent contact with confidants and higher proportion of kin. As mental health improves, the average frequency of contact increases. No significant difference in network change has been found between those older adults who worked for pay recently and those who did not. However, transitioning into working for pay is linked to more frequent contact with network members. The cohesiveness of the neighborhood in which older adults live also has implications for network change. Older adults who live in a more cohesive community tend to have a larger network size, higher frequency of contact, and lower proportion of kin. These results suggest that, despite pre-existing social disadvantage, positive contextual factors can lead to increase in network size, more contact with friends, and lower proportion of kin. Hypothesis 3 is partially supported. As for control variables, older adults who were married or had a partner present at Round 1 tend to have a higher proportion of kin than those who were not married or did not have a partner present. Those who transitioned into being married or having a partner present increased their proportion of kin in the confidant network. Older adults with a larger household size are likely to contact network members more often. Moreover, a greater proportion of these contacts are kin members.

## Conclusions and discussion

The changes in the size and structure of close social ties impact essential life outcomes of older adults (English and Carstensen 2014; Goldman and Cornwell 2018; Litwin and Levinson 2018). However, what leads to these changes is not yet fully understood. Socioemotional selectivity theory and social convoy theory claim close social ties are more enduring than peripheral or weak ties in the aging process. The core question these theories address is which type of social ties are more enduring throughout the life course. However, even the strongest ties dissolve or change over a long-time period (Cornwell et al. 2021). Moreover, the pattern of network change differs for different social groups. For instance, Suanet and Huxhold’s (2020) study on two Dutch cohorts suggests that the 1938–47 birth cohort is more likely to have an increase in network size around retirement age than the 1928–37 cohort, which is related to increased educational level and more diverse social roles. For another, the size and structure of the convoys of an adult are contingent on their own position in the network (Antonucci et al. 2014).What previous scholarship leaves unanswered is how the change in the strongest ties across time differs by socioeconomic status. To what extent do pre-existing social disadvantage and contextual factors shape one’s closest social network ties? Based on the longitudinal study of older adults egocentric networks, I find that the pattern of network change in later life is contingent on the social background and the contextual factors in which individuals are situated. Additionally, I did not observe a monotonic decrease in network size over the ten years as previous studies suggested.

My findings suggest that pre-existing social disadvantage is associated with less favorable network features for older adults. Older adults who did not attend college have fewer close friends with whom to discuss important matters and have a higher proportion of kin in their close social circles. Likewise, Black older adults and Hispanic older adults have fewer confidants and are more likely to have kin as their close friends. Some scholars argue that the rule of homophily based on race and ethnicity might contribute to a smaller network size for older Black adults (Vanhoutte and Hooghe 2012). However, studies have shown that homophily based on racial and ethnic background in the friendship network does not benefit racial minorities (Moody 2001). A larger and more diverse network often indicates more social resources and benefits are available from the network members (Litwin and Levinson 2018). However, whether an individual can build and maintain such advantageous networks largely depends on their socioeconomic status and pre-existing resources. These findings align with previous scholarship. In the meantime, I find that less educated and racial minority older adults have a higher frequency of contact with confidants, compared to their counterparts. This could be because these older adults are more reliant on their network members for support and resources on a daily basis. I propose that contacting network members frequently is a necessity and functions as a strategy to activate the potential resources in the network. Close social ties, in this case, act as a complementary resource to make up for the lack of financial, cultural, and political resources available for these older adults.

I claim that social contexts and the shifts in these contexts can alter personal networks in significant ways, even though pre-existing social disadvantage restrains the size and diversity of personal networks. For instance, neighborhood cohesion is positively associated with the network size and negatively associated with proportion of kin. The social capital at the community level can be transferred to an individual level. Older adults living in a cohesive community have more chances to meet people outside of their family and build connections. A welcoming community also encourages people to initiate contacts and stay connected. On the other hand, instability in a variety of key social and personal variables might cause shifts in the network and the pattern of network change (Goldman and Cornwell 2018). Also, I find that mental health is linked to the size and structure of the close social circle. Older adults with better mental health tend to contact their close friends more often. Transitioning into better mental health or transitioning into working status is associated with more frequent contact with confidants. Better mental health empowers older adults to reach out and activate their social capital. If retired older adults return to work, this can still ignite social contact and connections.

Based on these findings, I propose that older adults make efforts to adjust to changes in their networks during the aging process. While holding the social background constant, within-individual changes in personal networks can be seen as the consequence of individual’s efforts to cope with changes in the social context. Since a diverse and well-connected network is associated with more positive life outcomes (Litwin and Levinson 2018), older adults from disadvantaged backgrounds have more motivation to initiate contacts and stay connected. For instance, the death of a spouse is related to an increase in participation in group activities, frequency of contacting friends and family, and familial support (Iveniuk et al. 2020). For disadvantaged older adults, the close social circle can be the only resource to cope with negative life events. However, for privileged older adults, there can be multiple alternative resources at their disposal. Thus, these adults have less frequent need to contact their close ties. The cultivation and maintenance of social ties in later life depends not only on socioeconomic background, but also on propulsive action that not everyone can take. However, to what extent personal effort counts in making and sustaining social connections needs to be further examined. It takes a considerable amount of time, material resources, and emotional labor to maintain and convert social capital to materialistic or emotional resources. Individuals from disadvantaged social backgrounds have a higher likelihood of experiencing instability in the social environment and are more vulnerable to negative changes (Goldman and Cornwell 2018). When pre-existing disadvantage and less favorable social context compound, the stress they place on the social networks of the disadvantaged is also aggregated. In the end, it can lead to the concentration of social disadvantage.

This paper has a few limitations. First, this paper did not investigate the socioeconomic status of the network members of the older adults. Previous research suggests that older adults adjust networks in a way that favors their emotional needs over their materialistic needs. However, I propose that the way older adults utilize their network is highly likely to depend on their life situation and socioeconomic background. I mainly focus on the features of the ego and the contexts in which the ego is situated. I did not include any dyadic-level factors. Due to this limitation, I did not examine the network members’ social resources. To understand how much the confidant network could help older adults in daily life, one needs to know what resources these network members provide for the older adults. Frequent contact could offer the emotional support that older adults need. Financial assistance is also a critical aspect of networks as resources. However, the NSHAP dataset does not have information on the degree to which the confidants provide financial aid for older adults. I also do not have data on the general material support that older adults obtained from their confidants. But previous studies based on empirical data have repeatedly shown that networks can provide various resources. Therefore, I assume this as a given. For future research, it would be helpful to collect data on what type of resources the ego obtains from each social tie.

Second, this paper uses only three rounds of data across ten years to model change in network size and structure. That is to say, the survey may fail to capture changes in contextual factors and network features over short time scales. However, considering the size of the final sample, I still found significant association between the change in the social context and network features. Ideally, future research will benefit from more frequently collected longitudinal network data. Third, this paper only investigates the dynamics of older adults’ close ties. It does not show how the weaker ties change as adults age. The pattern of change in social ties could be very different for the stronger ties and the weaker ties. Finally, due to the limitations of the data, the aspects of key explanatory variables and their change that I can include in the models are limited. Further research along these lines will help to improve our understanding of the relationship between various contextual factors and network dynamics.

## Footnotes

1 The confidants include a variety of identities: Ex-spouse, Romantic/Sexual partner, Parent; Parent in-law; Child; Step-child; Brother or sister; Other relative of yours; Other in-law; Friend; Neighbor; Co-worker or boss; Minister, priest, or other clergy; Psychiatrist, psychologist, counselor, or therapist; Caseworker/Social worker; Housekeeper/Home health care provider; Other.

2 I assigned the scores to different mental health categories as below: 1 = poor, 2 = fair, 3 = good, 4 = very good, 5 = excellent. Higher scores indicate better mental health.

3 Similar to previous research, I assigned scores to each of nine functional health categories as follows: 0 = no difficulty, − 1 = some difficulty, − 2 = much difficulty, − 3 = unable to do. Scores closer to 0 indicate better functional health.

4 The race/ethnicity category in this paper is the same as the category in the original data from NSHAP. NSHAP divides race and ethnicity into four categories: 1) White, 2) Black, 3) Hispanic, non-Black, 4) Other.
